# Vaccination Rashes

**Published:** 1908-11-21

**Authors:** 


### DERMATOLOGY.

### VACCINATION RASHES.

In most cases vaccination has nothing to do with subsequent skin eruptions, although this truth is unfortunately not likely to commend itself to the lay mind. Possible non-adventitious rashes may be divided into two groups. First we have those arising from pure vaccine inoculation—secondary local eruptions of vaccinia occurring either before or after regular vesicle formation; in rare instances they may be generalised. Appearances of various forms merely due to irritation may also be noted. More important, from the point of view of prophylaxis, are the conditions arising from some admixture with the vaccine virus, either at the time of inoculation or secondarily after rupture of the vesicles. Impetigo and the lesions of tubercle or of syphilis are the three likely dangers in the former case. Carter states that the inoculation of tubercle is impossible with glycerinated calf lymph—and infection with syphilis should also be impossible.

It must be remembered in this connection that the irritation of vaccination may determine the outbreak of a congenital syphilitic rash: syphilis cannot be charged to vaccination unless its eruption occurs about fifty days after the operation has taken place. Glycerination is supposed to obviate also all risk of introduction of active pyogenic organisms. Sir Almroth Wright, however, found that vaccine from a calf whose resistance to staphylococcus he had raised by bacteriological methods, caused much less inflammation than the ordinary commercial article. This additional safeguard against sepsis, though, is clearly not very practicable. The occurrence of gangrene, erysipelas, or cellulitis from improper care after vesicular rupture is manifestly possible. The rashes remaining for consideration may, according to the view taken of their ætiology, be placed in either of the two main groups above indicated. The most common are roseola; a scarlatiniform or morbilliform erythema, as late as the eighteenth day; and a lichen, consisting of papules invading the whole body in successive crops, and generally showing at the end of the first week. Care in selecting lymph, asepsis, and cleanliness are the three essentials in prophylaxis.

